# Supplementary material for: Pyruvate Dehydrogenase Kinase 1 inhibition mediated oxidative phosphorylation enhancement in cartilage promotes osteoarthritis progression
Source: BMC Musculoskelet Disord. 2023 Jul 20;24:597. doi: 10.1186/s12891-023-06585-6 (PMC10357736; doi:10.1186/s12891-023-06585-6)
Supplement: Supplementary file 1 — Additional file 1. [file 12891_2023_6585_MOESM1_ESM.zip › BP.Gsea.1653623667859/GOBP_MRNA_SPLICE_SITE_SELECTION.html]

Details for gene set GOBP\_MRNA\_SPLICE\_SITE\_SELECTION[GSEA]

|  || Dataset | 55457\_collapsed\_to\_symbols.cls.cls #OA\_versus\_normal.cls.cls #OA\_versus\_normal\_repos |
| Phenotype | cls.cls#OA\_versus\_normal\_repos |
| Upregulated in class | normal |
| GeneSet | GOBP\_MRNA\_SPLICE\_SITE\_SELECTION |
| Enrichment Score (ES) | -0.58225864 |
| Normalized Enrichment Score (NES) | -1.9079099 |
| Nominal p-value | 0.0018018018 |
| FDR q-value | 0.056053475 |
| FWER p-Value | 0.689 |
Table: GSEA Results Summary

  

Fig 1: Enrichment plot: GOBP\_MRNA\_SPLICE\_SITE\_SELECTION      
 Profile of the Running ES Score & Positions of GeneSet Members on the Rank Ordered List

  

| SYMBOL | TITLE | RANK IN GENE LIST | RANK METRIC SCORE | RUNNING ES | CORE ENRICHMENT || 1 | SRSF9 | serine and arginine rich splicing factor 9 [Source:HGNC Symbol;Acc:HGNC:10791] | 882 | 0.548 | -0.0139 | No |
| 2 | PSIP1 | PC4 and SFRS1 interacting protein 1 [Source:HGNC Symbol;Acc:HGNC:9527] | 1482 | 0.431 | -0.0175 | No |
| 3 | SF3A1 | splicing factor 3a subunit 1 [Source:HGNC Symbol;Acc:HGNC:10765] | 3152 | 0.235 | -0.1237 | No |
| 4 | PUF60 | poly(U) binding splicing factor 60 [Source:HGNC Symbol;Acc:HGNC:17042] | 3237 | 0.227 | -0.1076 | No |
| 5 | SNRPC | small nuclear ribonucleoprotein polypeptide C [Source:HGNC Symbol;Acc:HGNC:11157] | 3649 | 0.190 | -0.1206 | No |
| 6 | SF3A2 | splicing factor 3a subunit 2 [Source:HGNC Symbol;Acc:HGNC:10766] | 5999 | -0.004 | -0.3026 | No |
| 7 | NOL3 | nucleolar protein 3 [Source:HGNC Symbol;Acc:HGNC:7869] | 7545 | -0.122 | -0.4104 | No |
| 8 | SETX | senataxin [Source:HGNC Symbol;Acc:HGNC:445] | 8398 | -0.185 | -0.4582 | No |
| 9 | KHDC4 | "KH domain containing 4, pre-mRNA splicing factor [Source:HGNC Symbol;Acc:HGNC:29145]" | 9997 | -0.321 | -0.5503 | Yes |
| 10 | SRSF1 | serine and arginine rich splicing factor 1 [Source:HGNC Symbol;Acc:HGNC:10780] | 10272 | -0.348 | -0.5369 | Yes |
| 11 | SRSF5 | serine and arginine rich splicing factor 5 [Source:HGNC Symbol;Acc:HGNC:10787] | 10282 | -0.349 | -0.5028 | Yes |
| 12 | SF3A3 | splicing factor 3a subunit 3 [Source:HGNC Symbol;Acc:HGNC:10767] | 10311 | -0.352 | -0.4699 | Yes |
| 13 | LUC7L2 | "LUC7 like 2, pre-mRNA splicing factor [Source:HGNC Symbol;Acc:HGNC:21608]" | 10334 | -0.354 | -0.4364 | Yes |
| 14 | LUC7L3 | LUC7 like 3 pre-mRNA splicing factor [Source:HGNC Symbol;Acc:HGNC:24309] | 10647 | -0.388 | -0.4220 | Yes |
| 15 | SRSF6 | serine and arginine rich splicing factor 6 [Source:HGNC Symbol;Acc:HGNC:10788] | 10659 | -0.389 | -0.3841 | Yes |
| 16 | YTHDC1 | YTH domain containing 1 [Source:HGNC Symbol;Acc:HGNC:30626] | 10687 | -0.391 | -0.3472 | Yes |
| 17 | CELF2 | CUGBP Elav-like family member 2 [Source:HGNC Symbol;Acc:HGNC:2550] | 10832 | -0.409 | -0.3176 | Yes |
| 18 | SRSF10 | serine and arginine rich splicing factor 10 [Source:HGNC Symbol;Acc:HGNC:16713] | 10926 | -0.422 | -0.2829 | Yes |
| 19 | CELF3 | CUGBP Elav-like family member 3 [Source:HGNC Symbol;Acc:HGNC:11967] | 11510 | -0.502 | -0.2781 | Yes |
| 20 | LUC7L | LUC7 like [Source:HGNC Symbol;Acc:HGNC:6723] | 11627 | -0.521 | -0.2352 | Yes |
| 21 | SF1 | splicing factor 1 [Source:HGNC Symbol;Acc:HGNC:12950] | 11664 | -0.527 | -0.1855 | Yes |
| 22 | PRPF39 | pre-mRNA processing factor 39 [Source:HGNC Symbol;Acc:HGNC:20314] | 11854 | -0.560 | -0.1444 | Yes |
| 23 | PTBP2 | polypyrimidine tract binding protein 2 [Source:HGNC Symbol;Acc:HGNC:17662] | 12211 | -0.649 | -0.1073 | Yes |
| 24 | CELF1 | CUGBP Elav-like family member 1 [Source:HGNC Symbol;Acc:HGNC:2549] | 12391 | -0.704 | -0.0511 | Yes |
| 25 | SFSWAP | splicing factor SWAP [Source:HGNC Symbol;Acc:HGNC:10790] | 12747 | -0.908 | 0.0119 | Yes |
Table: GSEA details [plain text format]

  

Fig 2: GOBP\_MRNA\_SPLICE\_SITE\_SELECTION      
 Blue-Pink O' Gram in the Space of the Analyzed GeneSet

  

Fig 3: GOBP\_MRNA\_SPLICE\_SITE\_SELECTION: Random ES distribution      
 Gene set null distribution of ES for **GOBP\_MRNA\_SPLICE\_SITE\_SELECTION**
